# Attenuating immune pathology using a microbial-based intervention in a mouse model of cigarette smoke-induced lung inflammation

**DOI:** 10.1186/s12931-017-0577-y

**Published:** 2017-05-15

**Authors:** Mark Bazett, Agnieszka Biala, Ryan D. Huff, Matthew R. Zeglinksi, Philip M. Hansbro, Momir Bosiljcic, Hal Gunn, Shirin Kalyan, Jeremy A. Hirota

**Affiliations:** 1 Qu Biologics Inc., Vancouver, BC Canada V5T 4T5; 2 Department of Medicine, Division of Respiratory Medicine, University of British Columbia, Vancouver, BC Canada V6H 3Z6; 3 iCORD Research Centre, University of British Columbia, Vancouver, BC Canada V5Z 1M5; 4 Priority Research Centre for Healthy Lungs, Hunter Medical Research Institute, The University of Newcastle, Newcastle, NSW Australia; 5 Department of Medicine, Division of Endocrinology, CeMCOR, University of British Columbia, Vancouver, BC Canada V5Z 1M9; 6 Firestone Institute for Respiratory Health, Division of Respirology, Department of Medicine, McMaster University, Hamilton, ON Canada L8N 4A6

**Keywords:** COPD, Immunomodulators, *Klebsiella*, mucosal immunology

## Abstract

**Background:**

Cigarette smoke exposure is the major risk factor for developing COPD. Presently, available COPD treatments focus on suppressing inflammation and providing bronchodilation. However, these options have varying efficacy in controlling symptoms and do not reverse or limit the progression of COPD. Treatments strategies using bacterial-derived products have shown promise in diseases characterized by inflammation and immune dysfunction. This study investigated for the first time whether a novel immunotherapy produced from inactivated *Klebsiella* (hereafter referred to as KB) containing all the major *Klebsiella* macromolecules, could attenuate cigarette smoke exposure-induced immune responses. We hypothesized that KB, by re-directing damaging immune responses, would attenuate cigarette smoke-induced lung inflammation and bronchoalveolar (BAL) cytokine and chemokine production.

**Methods:**

KB was administered via a subcutaneous injection prophylactically before initiating a 3-week acute nose-only cigarette smoke exposure protocol. Control mice received placebo injection and room air. Total BAL and differential cell numbers were enumerated. BAL and serum were analysed for 31 cytokines, chemokines, and growth factors. Lung tissue and blood were analysed for Ly6C^HI^ monocytes/macrophages and neutrophils. Body weight and clinical scores were recorded throughout the experiment.

**Results:**

We demonstrate that KB treatment attenuated cigarette smoke-induced lung inflammation as shown by reductions in levels of BAL IFNγ, CXCL9, CXCL10, CCL5, IL-6, G-CSF, and IL-17. KB additionally attenuated the quantity of BAL lymphocytes and macrophages. In parallel to the attenuation of lung inflammation, KB induced a systemic immune activation with increases in Ly6C^HI^ monocytes/macrophages and neutrophils.

**Conclusions:**

This is the first demonstration that subcutaneous administration of a microbial-based immunotherapy can attenuate cigarette smoke-induced lung inflammation, and modulate BAL lymphocyte and macrophage levels, while inducing a systemic immune activation and mobilization. These data provide a foundation for future studies exploring how KB may be used to either reverse or prevent progression of established emphysema and small airways disease associated with chronic cigarette smoke exposure. The data suggest the intriguing possibility that KB, which stimulates rather than suppresses systemic immune responses, might be a novel means by which the course of COPD pathogenesis may be altered.

**Electronic supplementary material:**

The online version of this article (doi:10.1186/s12931-017-0577-y) contains supplementary material, which is available to authorized users.

## Background

Chronic obstructive pulmonary disease (COPD) is an inflammatory airway disease that results in progressive irreversible airflow limitation. The global prevalence of COPD, as determined by the World Health Organization, is 11.7% [1], and it is predicted that by 2030 it will be the fourth leading cause of death worldwide [2]. Mainstream or second-hand cigarette smoke exposure is a risk factor for developing COPD [3], which can be exacerbated by genetic factors [4, 5] and environmental exposures [6, 7]. Chronic cigarette smokers that develop COPD may present with varying degrees of cough, sputum production, dyspnea, wheezing, and chest tightness [8, 9]. Presently, available treatments primarily focus on suppressing inflammation and providing bronchodilation. However, these options have varying efficacy in controlling symptoms and do not reverse or limit, completely, the progression of COPD [10, 11].

The pathology of COPD includes emphysema and obstruction of the small airways as a result of chronic bronchitis, which is associated with inflammation and immune dysfunction [12, 13]. In COPD patients, the inflammatory immune response is altered, and often involves increased cytokines, including IFN-γ, CXCL9 (MIG), CXCL10 (IP-10), and CCL5 (RANTES) [14–16]. This lung cytokine and chemokine milieu recruits and activates inflammatory cells including neutrophils, macrophages, B cells, CD4+ T cells, and CD8+ T cells [17, 18]. In addition to inflammation, abnormal immune function has also been described in COPD patients, including altered macrophage function [19]. Treatment options that can target the immune skewing and dysfunction, rather than broad immune repression, may present a more attractive approach to manage cigarette smoke-induced COPD.

Treatments strategies using bacterial-derived products have shown promise in diseases characterized by inflammation and immune dysfunction [20–23]. This has primarily been demonstrated in animal models of allergic airway disease where different treatment strategies using bacteria or bacterial derived products have been used to modulate immune responses [20–22, 24]. Towards that end, intervention strategies encompassing everything from live bacteria to specific pattern recognition receptor agonists have been used in models of allergic airway disease [21, 22, 25–27]. The apparent mechanism of action works through a reduction in inflammation and an altering of the immune response. In studies of smoking-induced lung disease, *Lactobacillus rahmnosus* and *Bifidobacterium breve* have been shown to attenuate pro-inflammatory cytokine production in a macrophage cell line treated with cigarette smoke extract [28].

The current study investigated for the first time whether a novel immunotherapy produced from inactivated *Klebsiella* (hereafter referred to as KB) containing all the major *Klebsiella* macromolecules, would attenuate maladaptive cigarette smoke exposure-induced immune responses. We hypothesized that KB would re-direct damaging immune responses and attenuate cigarette smoke-induced lung cellular inflammation and bronchoalveolar lavage (BAL) cytokine and chemokine production. We demonstrate that subcutaneous administration of KB attenuated cigarette smoke-induced lung inflammation and the quantity of airway BAL lymphocytes and macrophages while inducing systemic immune activation mimicking the response to acute infection.

## Methods

### Animals

Female mice C57BL/6 age 8–10 weeks old were purchased (Jackson Labs, Farmington, Connecticut, USA), acclimatized, and housed for one additional week prior to the commencement of experiments. Female mice were used as recent studies suggest that they are more susceptible to cigarette smoke induced lung pathology, as are women compared to men [29]. The experiments included ten mice per group, which were housed as five mice per cage in environmentally-controlled specific pathogen free conditions with a 12:12 h light/dark cycle for the duration of the study. All protocols were reviewed and approved by the Animal Care Committee of the University of British Columbia (Vancouver, BC, Canada).

### Cigarette/air smoking protocol

**Fig. 1:**
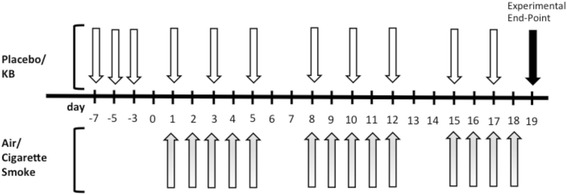
Cigarette smoke exposure protocol with *Klebsiella* (KB) intervention. Four groups of mice were exposed to either placebo + room air, KB + room air, placebo + cigarette smoke, or KB + cigarette smoke. *Grey* arrows, room air or cigarette smoke;* white* arrows, subcutaneous injection of placebo or KB. See methods for details

Air or cigarette smoke exposure was done for five consecutive days for the first 2 weeks and for four consecutive days in the third week (experimental days: 1–5; 8–12, 15–18, Fig. 1). Mice were euthanized 24 h after the last exposure (experimental day 19). Briefly, cigarette smoke exposure (University of Kentucky Research Grade Cigarettes) was performed by placing mice into plexiglass “nose only” exposure chambers as previously described [29, 30]. Each mouse smoked three cigarettes per day for a total of 45 min of exposure. Control room air-exposed mice were restrained for a similar duration without exposure to smoke. Animals were monitored throughout the smoke exposure procedure and for an additional 30 min post-smoke exposure.

### Microbe-based intervention strategy

The microbe-based intervention, KB, is a proprietary immunomodulator consisting of all major macromolecules of an inactivated pathogenic *Klebsiella* strain was originally isolated from a patient with acute pneumonia. KB was supplied by Qu Biologics (Vancouver, BC). For the treatment intervention, KB or a placebo vehicle control (physiological saline containing 0.4% phenol) was prophylactically administered on the experimental day −7, −5, −3, and the regimen continued throughout the experiment on days 1, 3, 5, 8, 10, 12, 15, 17 (Fig. 1). Each administration involved a subcutaneous injection of 30 μL of placebo or KB, which was alternatively delivered into the lower right abdomen, the lower left abdomen, the upper right chest, and the upper left chest, rotating clockwise for each injection day.

### Blood collection, BAL, and cytospin analysis of BAL cell differentials

Processing and analysis of collected terminal blood and BAL samples was done as described previously [22]. Cytospins were performed and cells in BAL evaluated based on morphology and Wright-Giemsa staining. BAL cell differentials were then counted using the prepared cytospin slide with 100 cells per mouse counted in a blinded fashion.

### Immune mediator profiling of BAL and serum samples

Soluble mediator analysis in BAL and serum was performed using a 31 cytokine, chemokine, growth factor multiplex kit according to the manufacturer’s protocol (Millipore, St. Charles, MO, USA) using the Bio-Plex™ 200 system (Bio-Rad Laboratories, Inc., Hercules, CA, USA). The multiplex was performed by Eve Technologies (Eve Technologies Corp, Calgary, AB, Canada). The 31-plex assay included the following mediators: Eotaxin, G-CSF, GM-CSF, IFNγ, IL-1α, IL-1β, IL-2, IL-3, IL-4, IL-5, IL-6, IL-7, IL-9, IL-10, IL-12 (p40), IL-12 (p70), IL-13, IL-15, IL-17, IP-10 (CXCL10), KC (CXCL1), LIF, MCP-1 (CCL2), M-CSF, MIG (CXCL9), MIP-1α (CCL3), MIP-1β (CCL4), MIP-2 (CXCL2), RANTES (CCL5), TNFα, and VEGF. The assay sensitivities of these markers range from 0.1 - 33.3 pg/mL.

### Flow cytometric analysis of Ly6C^HI^ monocytes/macrophages and neutrophils

Blood was collected in EDTA coated tubes (BD Microtainer) to prevent clotting and stored on ice prior to staining. Anti-coagulant-treated whole blood was stained with antibodies (CD11b-FITC, Ly6G-PE, CD11c-PerCPCy5.5 and Ly6C-APC) before red blood cell lysis (BD lysis buffer). Flow cytometry was run on a FACSCalibur (BD Bioscience). Analysis was completed using the FlowJo V10.1 program. Neutrophils were defined as Ly6G^+^CD11b^+^ cells. Ly6C^HI^ monocytes/macrophage were defined as Ly6C^HI^Ly6G^−^CD11b^+^ cells. Lymphocyte populations were gated on by forward scatter and side scatter and then defined as B220^+^, CD3^+^CD4^+^, or CD3^+^CD8^+^.

### Data analysis

GraphPad Prism 6 Software (GraphPad Software, San Diego, CA) was used to perform statistical analysis of the results. Data are expressed as mean ± SD. One-way ANOVA analysis followed by multiple comparisons using a Sidak post-hoc test was performed for group comparisons. Four experimental group combinations were compared; room air-placebo vs. room air-KB, room air-placebo vs. cigarette smoke-placebo, room air-KB vs. cigarette smoke-KB, cigarette smoke-placebo vs. cigarette smoke-KB. Differences were reported as statistically significant when *p* < 0.05.

## Results

### KB attenuated cigarette smoke exposure-induced influx of lymphocytes and macrophages, but not neutrophils into the airways

**Fig. 2:**
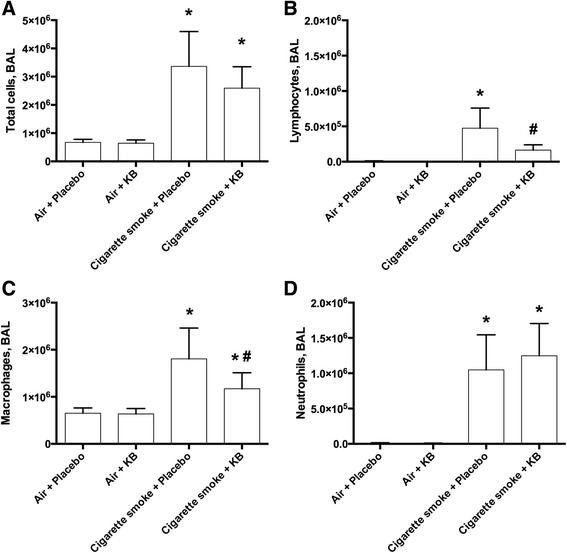
KB treatment attenuated cigarette smoke exposure induced increases in airway macrophages and lymphocytes but not total cells or neutrophils. BAL cell counts and differentials following placebo and KB treatment in room air or cigarette smoke-exposed groups. **a** BAL total cells, **b** lymphocytes, **c**, macrophages **d**, and neutrophils. * *p* < 0.05 comparing to the groups relative control; # *p* < 0.05 comparing KB group to relative placebo control. Data are means ± SD of 9–10 mice per group

A three-week acute model of cigarette smoke exposure in mice (Fig. 1) was used to investigate how KB exposure can modulate lung inflammation. The total BAL cell counts and cellular differentials for each experimental group were assessed (Fig. 2). In placebo treated animals, cigarette smoke exposure increased the total number of BAL cells (Fig. 2a, *p* < 0.0001). The observed increase in cellularity resulted from increases in the numbers of lymphocytes, macrophages, and neutrophils (Fig. 2b, d, *p* < 0.0001), but not eosinophils (*p* = 0.35, data not shown). KB intervention did not significantly decrease the total number of BAL cells, however it did attenuate the increase in lymphocytes and macrophages in the cigarette smoke-exposed group (*p* < 0.005, Fig. 2b-c) while having no detectable impact on neutrophils (Fig. 2d, *p* = 0.59).

### KB intervention attenuated cigarette smoke exposure-induced lung inflammatory responses

**Fig. 3:**
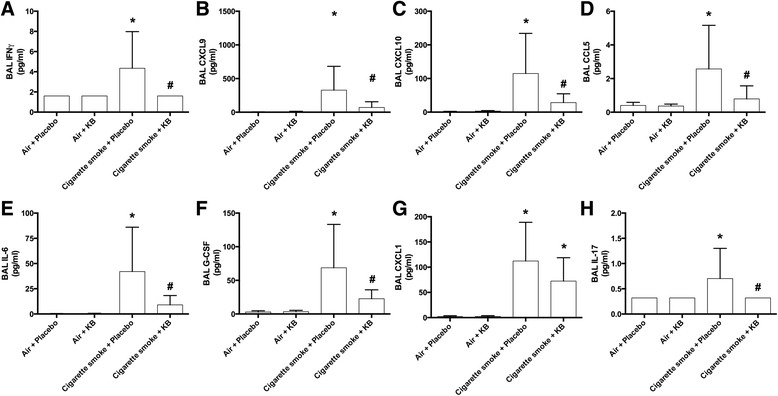
KB treatment attenuated cigarette smoke exposure induced increases Th1-skewed lung inflammatory responses. BAL supernatant fluid analysis following placebo and KB treatment in room air or cigarette smoke-exposed groups. **a** IFNγ, **b** CXCL9, **c** CXCL10, **d** CCL5, **e** IL-6, **f** G-CSF, **g** CXCL1, **h** IL-17. * *p* < 0.05 comparing to the groups relative control; # *p* < 0.05 comparing KB group to relative placebo control. Data are means ± SD of 10 mice per group

Previous reports have demonstrated that cigarette smoke exposure models are characterized by a cytokine profile that includes IFN-γ, CXCL9 (MIG), CXCL10 (IP-10), and CCL5 (RANTES) [14, 15]. A multiplex analysis of 31 cytokines, chemokines, and growth factors (Additional file 1: Table S1) was used to investigate cytokine and chemokine production induced by cigarette smoke exposure in this experimental system. KB intervention had no impact on air-exposed animals for any mediator measured in the BAL fluid. Cigarette smoke exposure induced 15 of the 31 measured mediators in the BAL fluid, which were IFNγ, CXCL9, CXCL10, CCL5, IL-6, IL-17, G-CSF, CXCL1, LIF, CCL2, CCL3, CCL4, TNFα, eotaxin, and VEGF (*p* < 0.05). Although IL-17 was elevated with cigarette smoke exposure, this was only observed in 4 of 10 samples and the values were close to the level of detection for this cytokine (0.64 pg/ml). KB intervention attenuated cigarette smoke-induced increases in IFNγ, CXCL9, CXCL10, CCL5, IL-6, G-CSF, and IL-17 (Fig. 3, *p* < 0.05) in the BAL fluid. KB also decreased TNFα levels (cigarette smoke + placebo 7.50 ± 5.98 pg/ml vs cigarette smoke + KB 3.52 ± 3.34 pg/ml), but this was not statistically significant (*P* = 0.057, Additional file 1: Table S1).

### Systemic immune cytokine, chemokine and growth factor profile were not significantly altered with 3-week cigarette smoke exposure, but were augmented by KB exposure

**Fig. 4:**
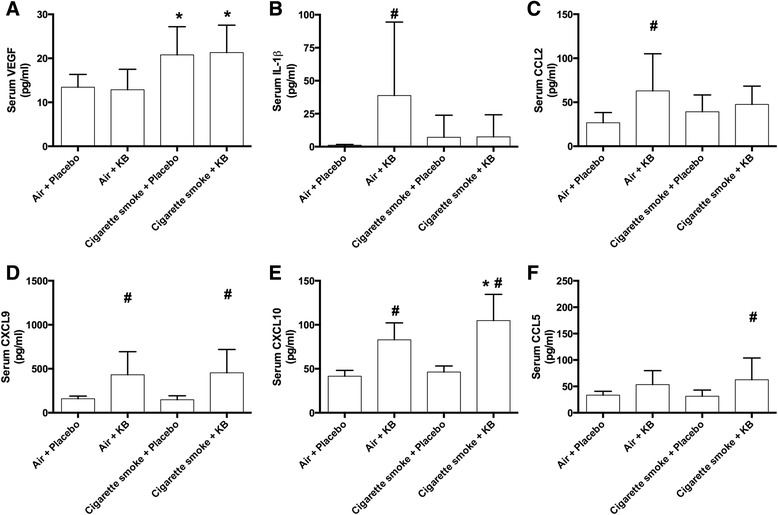
KB treatment differentially modulates cigarette smoke exposure induced changes in serum immune mediators. Serum analysis following placebo and KB treatment in room air or cigarette smoke-exposed groups. **a** VEGF, **b** IL-1β, **c** CCL2, **d** CXCL9, **e** CXCL10 and **f** CCL5. * *p* < 0.05 comparing to the groups relative control; # *p* < 0.05 comparing KB group to relative placebo control. Data are means ± SD of 9–10 mice per group

When assessing changes in the serum levels of cytokine, chemokines and growth factors in the experimental treatment groups, it was found that cigarette smoke exposure induced an increase in only VEGF, and this was not changed with the KB intervention (Fig. 4a, *p* < 0.05; Additional file 2: Table S2). KB treatment in air-exposed animals decreased only one mediator, IL12p40, while increasing serum levels of IL-1β, CCL2, CXCL9, and CXCL10 (Fig. 4b-d, *p* < 0.05). In the cigarette smoke-exposed mice, treatment increased the levels of CXCL9, CXCL10, and CCL5 relative to cigarette smoke + placebo treated groups (*p* < 0.05). Collectively these data suggest that KB intervention induced systemic immune responses that are independent of cigarette smoke exposure, which may play a role in the observed local down-regulation of cigarette smoke exposure-induced lung inflammation.

### KB induced both a systemic and local lung tissue increase in the proportion of Ly6C^HI^ monocytes/macrophages and neutrophils, with no change in lymphocyte populations

**Fig. 5:**
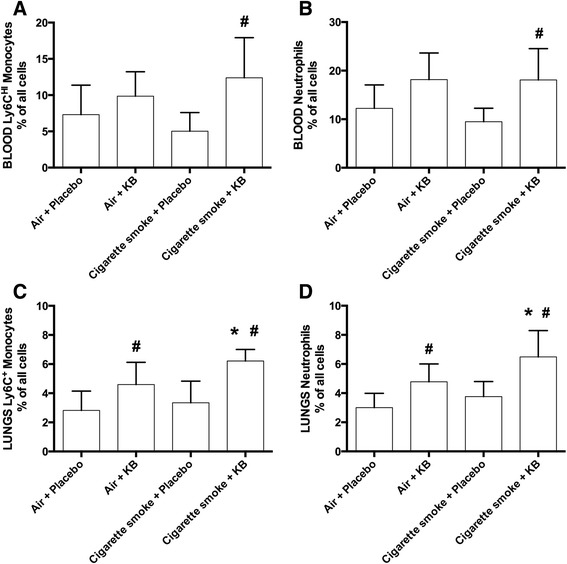
KB treatment increased blood and lung Ly6C^HI^ monocytes and neutrophils. Flow cytometric analysis of blood **a-b** and lung **c-d** Ly6C^HI^ monocytes and neutrophils following placebo and KB treatment in room air or cigarette smoke-exposed groups. * *p* < 0.05 comparing to the groups relative control. # *p* < 0.05 comparing KB group to relative placebo control. Data are means ± SD of 10 mice per group

To investigate if the KB intervention altered systemic or local cellular immune profiles, blood and lung cells were assessed by flow cytometry, with particular focus on the levels of Ly6C^HI^ monocytes (an inflammatory subgroup of monocytes, characterized as Ly6G^−^CD11b^+^ cells) and neutrophils (characterized as Ly6G^+^CD11b^+^ cells). Cigarette smoke exposure had no effects on the numbers of blood Ly6C^HI^ monocytes or neutrophils (Fig. 5a-b, *p* > 0.6). KB intervention increased the blood Ly6C^HI^ monocytes and neutrophils in the cigarette smoke exposure groups (*p* < 0.005), and the neutrophils in the air-exposed animals (*p* = 0.05). The increases in systemic Ly6C^HI^ monocytes and neutrophils were associated with local increases in the lung tissue (Fig. 5c-d), where KB induced increases in these cell types, which was further enhanced by cigarette smoke exposure (*p* < 0.05).

**Fig. 6:**
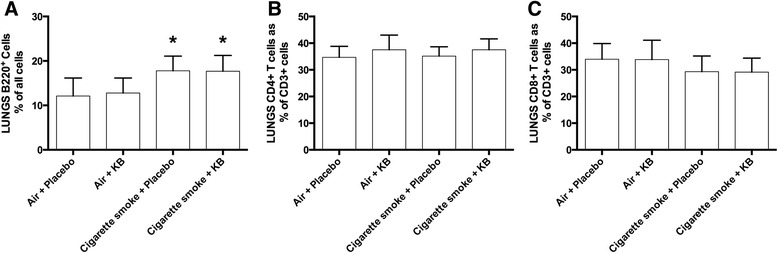
KB treatment has no impact on lung B220+ cells, CD3 + CD4+, or CD3 + CD8+ T cells. Flow cytometric analysis of lung **a** B220+ B cells, **b** CD3 + CD4+ T cells, or **c** CD3 + CD8+ T cells following placebo and KB treatment in room air or cigarette smoke-exposed groups. * *p* < 0.05 comparing to the groups relative control. Data are means ± SD of 10 mice per group

The levels of B and T lymphocytes were also assessed in the lungs. The percentage of B cells in the lungs after smoke exposure was elevated (Fig. 6a, *p* < 0.05), which was not attenuated by KB administration. No statistically significant change in the percentage of CD3^+^CD4^+^ or CD3^+^CD8^+^ cells was observed following cigarette smoke exposure or KB administration (Fig. 6b-c).

### Intervention with KB had no impact on clinical score and body weight following cigarette smoke exposure

**Fig. 7:**
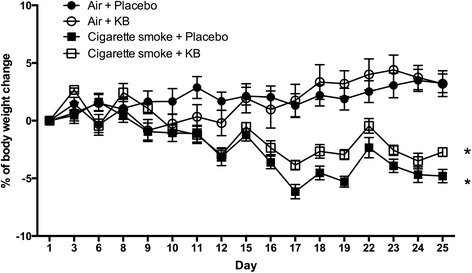
Cigarette smoke exposure impaired body weight gain independent of KB treatment. Daily measurements of mice were normalized to starting weight for each of the four groups. * *p* < 0.05 comparing to the group’s relative control. Data are means ± SD of 10 mice per group

Body weight and clinical score was used to monitor the overall health of mice exposed to cigarette smoke in the presence or absence of KB. Body weight was normalized to the starting weight of each animal and all animals were observed throughout the experiment and their health assessed based on a clinical score (e.g. hunched posture, interaction with other animals, activity levels). KB administration in the air-exposed group did not significantly alter the body weight (Fig. 7) nor impact the clinical score (data not shown). No adverse effects of repeated KB administration were observed. Cigarette smoke-exposed mice had a prominent loss in body weight (*p* < 0.05), which KB intervention did not attenuate (*p* > 0.05). Cigarette smoke exposure did not significantly changed the clinical score for either placebo or KB-treated mice.

## Discussion

There is growing awareness that exposure to microbial products can alter the course of inflammatory diseases. In asthma, several studies have demonstrated promising results for resolution of symptoms with microbial products in both animal models and clinical studies [21, 22, 24–27]; however, there is a paucity of data looking into the use of these approaches aimed at modulating the course of the immune dysfunction in COPD [31]. In this study, we tested the hypothesis that KB, which was produced from a clinical *Klebsiella* isolate containing all the major *Klebsiella* macromolecules, could modulate airway inflammation and immune responses in a mouse model of acute cigarette smoke exposure. These results demonstrate that prophylactic KB treatment attenuated both cigarette smoke-induced lung inflammation and BAL macrophage and lymphocyte cellularity. In control room air-exposed and experimental cigarette smoke-exposed animals, KB induced systemic immune responses, resulting in mobilization of monocytes and neutrophils. This systemic immune modulation was mirrored locally in lung tissue, reflected by an increase in Ly6C^HI^ monocytes/macrophages and neutrophils. These data therefore suggest that interventions with microbial components that *enhance* rather than suppress immune responses may provide a novel strategy to alter the course of cigarette smoke exposure related COPD pathogenesis. Future therapeutic intervention-dosing strategies will be aimed at determining how late in the course of smoke-induced lung damage such a microbe-based intervention strategy can be administered to reverse pathology.

Chronic cigarette smoke exposure in humans is associated with emphysema and chronic bronchitis in COPD patients. Acute cigarette smoke exposure can lead to inflammatory responses that may be important preceding events in the chronic changes to lung physiology [32]. The mouse model of acute cigarette smoke exposure used in this study was designed to determine the impact of KB on modulating these earlier alterations in the inflammatory response and not the chronic bronchitic or emphysematous phenotype observed in chronic mouse cigarette smoke exposure models [29, 33–38]. This study therefore focused on outcome measurements that are impacted by acute cigarette smoke exposure, including lung inflammation resulting from smoke-induced tissue damage, systemic inflammation, immune cell activation, and body weight.

Cigarette smoke exposure-induced inflammation has been described as T_H_1 skewed, although this may be an over simplification [14, 15]. Changes in inflammatory mediators are accompanied by elevations in macrophages, lymphocytes, and neutrophil populations [17, 18, 32]. IFN-γ has been implicated as an important participant in the development of emphysematous lesions following cigarette smoke exposure in mice [16] and has been associated with COPD in humans [14, 15]. IFN-γ induces the CXCR3 ligands, CXCL9 (MIG) and CXCL10 (IP-10) [39] and the CCR5 ligand, CCL-5 (RANTES) [16] which recruit lymphocytes and macrophages to sites of inflammation. Importantly, blocking this response protects mice from the pathological impacts of cigarette smoke exposure [16, 40]. KB intervention was found to specifically attenuate cigarette smoke-induced elevations in IFN-γ, CXCL9, CXCL10, CCL-5, and IL-6 in the BAL, which was uncoupled from systemic immune activation. This reduction in BAL inflammatory mediators was associated with a concomitant reduction in macrophage and lymphocyte recruitment to the airways. Future mechanistic studies are required to determine how modulation of systemic immune function alters the progression of lung immunity important in chronic models of cigarette smoke exposure.

COPD pathology has many pathologic pathways in common with other inflammatory diseases, including asthma and inflammatory bowel disease (IBD). In these indications, microbial products are actively under investigation as treatment options [23, 24, 31, 41]. IBD and COPD share common pathology relating to mucosal barrier disruption including an altered microbiome, immune dysfunction, altered epithelial cell function, and chronic inflammation [31, 41, 42]. Live microbial products are currently being tested for efficacy in IBD [43, 44]. Furthermore, a product that is prepared in a similar manner to KB, but produced from *Escherichia coli,* has shown evidence of efficacy in IBD patients [45] and is currently being investigated in clinical trials as a treatment for IBD. There is also significant overlap between asthma and COPD including altered respiratory microbiome and immune dysfunction [31, 42, 46, 47]. In animal models of allergic asthma, live bacteria and their components [24, 27, 48, 49], Toll-like receptor (TLR) agonists [50], and the KB product [22], have all reduced lung inflammation. Collectively, the primary research presented in this report and the studies outlined above demonstrate some of the similarities between COPD and other inflammatory diseases that benefit from microbial intervention strategies. Taken together, these findings suggest that enhancing or resetting the immune response with bacterial products could be a novel therapeutic approach to managing COPD.

Systemically, these data showed that KB administration, which contains all the major macromodules from the *Klebsiella,* increased certain cytokines conventionally considered as being pro-inflammatory cytokines, such as IL-1β, as well as the proportion of blood Ly6C^HI^ monocytes and neutrophils, similar to the response seen with an acute infection [51–54]. This immune activation and mobilization was also detected in the lung tissue by flow cytometry where an increase in the proportion of monocytes and neutrophils was observed. Conversely, the airways of KB treated animals showed a reduction in the macrophage and lymphocyte levels. This duality of the increased inflammation in the lung tissue and decreased inflammation in the airways highlights the importance of where the immune response occurs for resolution of symptoms. Although the precise mechanism(s) of this phenomenon are not yet clear, the prevailing evidence suggests that inflammatory monocytes can differentiate into multiple different cells types in inflamed/damaged tissue [19, 55, 56] and that enhancement of immune function in the correct tissue-microenvironment may paradoxically contribute to an attenuation in overall inflammation, potentially by clearing necrotic/damaged tissue and rebuilding the loss of barrier function. Indeed, the observed increase in the number of lung Ly6C^HI^/CD11b^+^ inflammatory monocytes may have the ability to suppress inflammation [56–58].

## Conclusions

Our study shows that KB, produced from a clinical *Klebsiella* isolate, can suppress the progression of local airway immune responses and lymphocyte and macrophage influx, while inducing a systemic inflammatory response, in a mouse model of acute cigarette smoke-induced lung inflammation. This is the first demonstration that subcutaneous administration of a microbial derived intervention, KB, can attenuate cigarette smoke-induced inflammation. These data provide a foundation for future studies exploring how KB may be used to either reverse or prevent progression of established emphysema and small airways disease associated with chronic cigarette smoke exposure. Lastly, the data suggest the intriguing possibility that KB, which stimulates rather than suppresses systemic immune responses, might be a novel means by which the course of COPD pathogenesis may be altered, highlighting the complex interaction between inflammation and COPD pathogenesis.

## Additional files

Additional file 1: Table S1. Soluble mediator analysis in BAL fluid following placebo and KB treatment in room air or cigarette smoke-exposed groups. * *p* < 0.05 comparing to the group’s relative control; # *p* < 0.05 comparing treated group to untreated relative control. *ns* = no significant difference. Data are means ± SD of 9–10 mice per group. (XLSX 39 kb)

Additional file 2: Table S2. Soluble mediator analysis in serum following placebo and KB treatment in room air or cigarette smoke-exposed groups. * *p* < 0.05 comparing to the group’s relative control; # *p* < 0.05 comparing treated group to untreated relative control. *ns* = no significant difference. Data are means ± SD of 9–10 mice per group. (XLSX 36 kb)

## Abbreviations

ANOVA: Analysis of variance
BAL: Bronchoalveolar lavage
COPD: Chronic obstructive pulmonary disease
EDTA: Ethylenediaminetetraacetic acid
G-CSF: Granulocyte-colony stimulating factor
GM-CSF: Granulocyte macrophage colony-stimulating factor
IFNγ: Interferon-gamma
LIF: Leukemia inhibitor factor
M-CSF: Macrophage colony-stimulating factor
TNFα: Tumor necrosis factor-alpha
VEGF: Vascular endothelial growth factor
IBD: Inflammatory bowel disease
TLR: Toll-like receptor
